# Preoxygenation before intubation in adult patients with acute hypoxemic respiratory failure: a network meta-analysis of randomized trials

**DOI:** 10.1186/s13054-019-2596-1

**Published:** 2019-09-18

**Authors:** Ka Man Fong, Shek Yin Au, George Wing Yiu Ng

**Affiliations:** Department of Intensive Care, Queen Elizabeth Hospital, 30 Gascoigne Road, Kowloon, Hong Kong SAR

**Keywords:** Respiratory failure, Noninvasive ventilation, High flow nasal cannula, Preoxygenation, Meta-analysis

## Abstract

**Background:**

Patients with acute hypoxemic respiratory failure are at risk for life-threatening complications during endotracheal intubation. Preoxygenation might help reduce the risk of hypoxemia and intubation-related complications. This network meta-analysis summarizes the efficacy and safety of preoxygenation methods in adult patients with acute hypoxemic respiratory failure.

**Methods:**

We searched PubMed, EMBASE, and the Cochrane Library Central Register of Controlled Trials through April 2019 for randomized controlled trials (RCT) that studied the use of conventional oxygen therapy (COT), high-flow nasal cannula (HFNC), noninvasive ventilation (NIV), and HFNC and NIV as preoxygenation before intubation in patients with acute hypoxemic respiratory failure. Citations’ screening, study selection, data extraction, and risk of bias assessment were independently performed by two authors. The primary outcome was the lowest SpO_2_ during the intubation procedure.

**Results:**

We included 7 RCTs (959 patients). Patients preoxygenated with NIV had significantly less desaturation than patients treated with COT (mean difference, MD 5.53, 95% CI 2.71, 8.34) and HFNC (MD 3.58, 95% CI 0.59, 6.57). Both NIV (odds ratio, OR 0.43, 95% CI 0.21, 0.87) and HFNC (OR 0.49, 95% CI 0.28, 0.88) resulted in a lower risk of intubation-related complications than COT. There were no significant mortality differences among the use of NIV, HFNC, COT, and HFNC and NIV during preoxygenation.

**Conclusions:**

In adult patients with acute hypoxemic respiratory failure, NIV is a safe and probably the most effective preoxygenation method.

## Background

Patients with acute hypoxemic respiratory failure are at increased risk for life-threatening complications during endotracheal intubation. Profound desaturation (SpO_2_ < 80%) during intubation was reported in 25% of patients [1]. Cardiac arrest occurred in 1 out of 40 intubations, and it was associated with hypoxemia and absence of preoxygenation [2]. Preoxygenation might help reduce the risk of hypoxemia and intubation-related complications.

Apart from conventional oxygen therapy (COT) delivered through bag-valve mask or face mask, noninvasive ventilation (NIV) and high-flow nasal cannula (HFNC) have been increasingly used in the intensive care units (ICU) as preoxygenation devices. Both HFNC [3–5] and NIV [6, 7] have been shown to offer better preoxygenation than COT. The recently published FLORALI-2 study comparing HFNC and NIV has added new insight to this battlefield [8].

Network meta-analysis (NMA) has been increasingly advocated in medical research [9]. Through a combination of direct and indirect estimates of effects, NMA allows comparison of multiple interventions and improved precision. The aim of this NMA is to evaluate the impact of preoxygenation, which includes desaturation during intubation, intubated-related complications, and mortality, by various devices including COT, HFNC, and NIV, in adults with acute hypoxemic respiratory failure.

## Methods

We adhered to the *Preferred Reporting Items for Systematic Reviews and Meta-analyses* extension statement for reporting network meta-analyses (PRISMA-NMA) (Additional file 1) [10]. The protocol for this review was registered in the International Prospective Register of Systematic Reviews (CRD42018085866).

### Data sources and searches

We searched PubMed, EMBASE, and the Cochrane Library Central Register of Controlled Trials through April 2019 for potentially relevant studies published in English. Our PubMed search strategy is presented in Additional file 2: Table S1. Reference lists of relevant articles were also reviewed. We included randomized controlled trials (RCT) of adult patients with acute hypoxemic respiratory failure investigating any form of preoxygenation devices during endotracheal intubation. Acute hypoxemic respiratory failure was defined by the individual authors in the included studies. Preoxygenation devices included COT via bag-valve mask or face mask, HFNC, or NIV. We defined preoxygenation as oxygen delivery during the period before induction of anesthesia, till initiation of laryngoscopy. Apneic oxygenation was defined as oxygen delivery to the nasopharynx during the time between initiation of laryngoscopy to the intubation of the trachea (Additional file 2: Figure S1). We excluded studies focusing only on apneic oxygenation. The following were excluded: studies evaluating only the duration of preoxygenation, decision on ventilation or preoxygenation during anesthesia or interventional procedures, or enrolling healthy volunteers or animals.

### Study selection and data extraction

Two authors (KF and SA) independently screened citations and abstracts in duplicate and independently. All references judged potentially relevant were evaluated for full-text eligibility. Discrepancies were solved by consensus with the third author (GN). When relevant data or information was missing, we attempted to contact authors of the studies.

### Outcome measures

The primary outcome was the lowest SpO_2_ during the intubation procedure (from beginning of laryngoscopy to confirmation of endotracheal intubation by capnography). The secondary outcomes were proportion of patients with severe desaturation (SpO_2_ < 80%), intubation-related complications (aspiration or new infiltrate on post-intubation chest radiograph, hemodynamic instability, and cardiac arrest), and mortality.

### Risk of bias assessment

Two authors (SA and GN) independently assessed the risk of bias of included studies. We assessed the risk of bias of RCTs using the revised Cochrane risk-of-bias tool for randomized trials [11]. In case of disagreement for the attribution of risk of bias, it was solved by discussion and consensus with the third author (KF).

### Statistical analysis and quality of evidence

We performed a random effect network meta-analysis using a frequentist framework, calculating mean differences (MD) for continuous outcomes and odds ratios (OR) for dichotomous outcomes. Where data were not available, we converted the median and interquartile range to mean and standard deviations using a published equation [12].

We used the package “netmeta” (version 1.0-1) in R (version 3.4.2, The R Foundation for Statistical Computing) to perform network meta-analysis [13]. The “netmeta” package is based on an approach that follows the graph-theoretical methodology. We ranked the treatment using the *P-*score which are based on the frequentist point estimates and their standard errors [14]. It represented the extent of certainty that a treatment is better than the other treatments—the *P*-score would be close to 1 when a treatment is certain to be the best and close to 0 when a treatment was certain to be the worst. Precision of the ranking is also taken into account by looking at confidence intervals. Homogeneity and consistency assumptions were checked using a generalized Cochrane’s Q statistics for multivariate meta-analysis [15]. Inconsistency in the random effect model was assessed by between-study *Q* statistic that was calculated based on design-by-treatment interaction model [16]. Sensitivity analysis was conducted by sequentially omitting one study each time.

We applied the modified *Grading of Recommendations Assessment, Development and Evaluation* (GRADE) approach for network meta-analysis [17, 18]. The contribution of all direct estimates to the network estimates was evaluated from the contribution matrix [19]. We would rate down the quality of evidence when intransitivity was present, or when there was incoherence between direct and indirect estimates. When both direct and indirect evidence were available, we chose the higher of the two quality ratings for the NMA estimate [17].

## Results

### Literature search

**Fig. 1:**
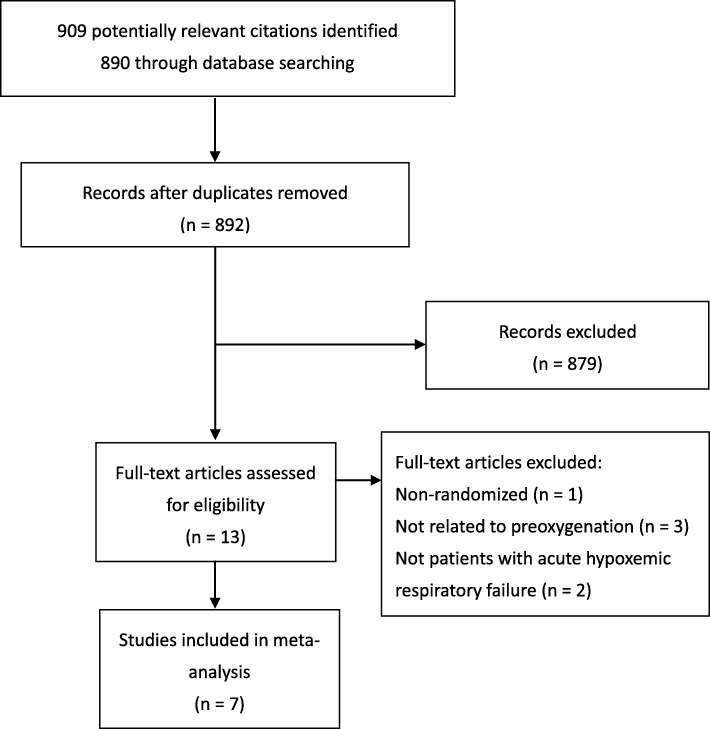
PRISMA flow diagram of the search results

The initial search yielded 909 citations; 13 proved potentially eligible after reviewing the full-text articles. Seven studies met our inclusion criteria, representing 959 patients (Fig. 1).

### Study characteristics

Of the 7 eligible trials, 3 compared HFNC with COT; 2, NIV with COT; 1, NIV with HFNC; and 1, HFNC and NIV with NIV (Table 1). Trial sample size ranged from 40 to 313 patients. Results of the individual studies could be found in Additional file 2: Tables S2–S3.

**Table 1:** Characteristics of the included studies

Study and published year	Settings	Participants	First intervention	Second intervention	PaO_2_ (mmHg) or PaO_2_/FiO_2_ ratio of the participants (mean ± SD or median [IQR])	Key outcomes
Baillard et al. [6] 2006	Two medical-surgical ICUs of 2 university hospitals in France	*N* = 53 Inclusion criteria: Acute respiratory failure requiring intubation Hypoxemia (PaO_2_ < 100 mmHg with 10 L/min O_2_ mask Exclusion criteria: encephalopathy or coma, cardiac resuscitation, hyperkalemia (> 5.5 mEq/L)	3-min preoxygenation with a nonrebreather bag-valve mask driven by 15 L/min O_2_ Patient allowed to breathe spontaneously with occasional assistance	3-min preoxygenation with NIV (PSV delivered by an ICU ventilator through a face mask adjusted to obtain an expired tidal volume of 7–10 ml/kg, FiO_2_ 100%, PEEP 5 cmH_2_O)	PaO_2_: COT, 68 [60–79] NIV, 60 [57–89]	Drop in SpO_2_ during endotracheal intubation Regurgitation, new infiltrate on post-procedural chest X-ray, SpO_2_ < 80% during intubation, ICU mortality
Vourc’h et al. [3] 2015	Six French ICUs (3 medical, 2 medical-surgical, one surgical)	*N* = 119 Inclusion criteria: Adults (≥ 18 years) with acute hypoxemic respiratory failure (RR > 30 bpm and FiO_2_ ≥ 50% to obtain > 90% oxygen saturation, and estimated PaO_2_/FiO_2_ < 300 mmHg) requiring endotracheal intubation in ICU after RSI Exclusion criteria: cardiac arrest, asphyxia, intubation without RSI, Cormack-Lehane grade 4 glottis	4-min preoxygenation with high FiO_2_ facial mask (15 L/min O_2_ flow)	4-min preoxygenation with HFNC set to 60 L/min, of humidified oxygen flow (FiO_2_ 100%); maintained in place throughout the endotracheal intubation	PaO_2_/ FiO_2_: Facial mask, 115.7 ± 63 HFNC, 120.2 ± 55.7	Lowest SpO_2_ during endotracheal intubation Incidence of desaturation SpO_2_ < 80%, cardiovascular collapse (SBP < 80 or vasopressor introduction or increasing doses more than 30%), aspiration, 28-day mortality
Jaber et al. [20] 2016	Single-center medical and surgical ICU in France	*N* = 49 Inclusion criteria: Patients with severe hypoxemic acute respiratory failure (RR > 30 bpm, FiO_2_ requirement ≥ 50% to obtain > 90% SpO2 or an impossibility to obtain > 90% SpO_2_, estimated PaO_2_/FIO_2_ < 300 mmHg) admitted to ICU requiring mechanical ventilation Exclusion criteria: cardiocirculatory arrest	4-min 30° head-up inclination with HFNC (humidified O_2_ flow 60 L/min, FiO_2_ 100%) combined with NIV (PS 10 cmH_2_O, PEEP 5 cmH_2_O, FiO_2_ 100%)	4-min 30° head-up inclination with NIV (PS 10 cmH_2_O, PEEP 5 cmH_2_O, FiO_2_ 100%)	PaO_2_/ FiO_2_: HFNC + NIV, 107 [74–264] NIV, 140 [83–201]	Minimal SpO_2_ during intubation, severe hypoxemia SpO_2_ < 80%, cardiovascular collapse (SBP < 65 mmHg at least once or < 90 mmHg lasting 30 min despite 500–1000 ml crystalloid loading or requiring introduction or increasing doses by more than 30% of vasoactive support), cardiac arrest, 28-day mortality
Simon et al. [4] 2016	Single center in Germany	*N* = 40 Inclusion criteria: Respiratory failure with hypoxemia (PaO_2_/FiO_2_ < 300 mmHg), indicated for endotracheal intubation, age ≥ 18 years Exclusion criteria Difficult airway, nasopharyngeal obstruction or blockage	3-min preoxygenation using a BVM (adult size AMBU SPUR II disposable resuscitator with oxygen bag reservoir and without PEEP valve or pressure manometer), O_2_ 10 L/min. No manual insufflation performed during apneic period.	3-min preoxygenation using HFNC, oxygen flow 50 L/min, FiO2 1.0; left in place during the intubation procedure	PaO_2_/ FiO_2_: BVM, 205 ± 59 HFNC, 200 ± 57	Lowest SpO_2_ during intubation, adverse events (cardiac arrest, arrhythmia, hemodynamic instability, aspiration of gastric contents)
Baillard et al. [7] 2018	Six sites in France	*N* = 201 Inclusion criteria: Adults patients (age > 18) with acute respiratory failure requiring intubation Exclusion criteria: Encephalopathy or coma, cardiac resuscitation, decompensation of chronic respiratory failure	3-min preoxygenation with non-rebreathing BVM with an oxygen reservoir driven by 15 L/min O_2_; patient allowed to breathe spontaneously with occasional assists	3-min preoxygenation using NIV—pressure support mode delivered by an ICU ventilator through a face mask adjusted to obtain an expired tidal volume of 6–8 ml/kg, FiO2 1.0, PEEP 5 cmH_2_O	PaO_2_/FiO_2_: BVM, 126 [95–207] HFNC, 132 [80–175]	Maximal value SOFA score within 7 days after intubation, requirement for an early stop of preoxygenation and immediate intubation, arrhythmia with hemodynamic failure, regurgitation, severe O_2_ desaturation SpO_2_ < 80%, 28-day mortality
Guitton et al. [5] 2019	Seven French ICU (4 medical, 2 medical-surgical, 1 surgical)	*N* = 184 Inclusion criteria: Adults patients (age > 18) requiring intubation in the ICU, without severe hypoxemia (PaO2/FiO2 < 200 mmHg) Exclusion criteria: Intubation without RSI (cardiac arrest), fiberoptic intubation, asphyxia, nasopharyngeal blockade, grade 4 glottis on Cormack-Lehane scale	4-min preoxygenation in a head-up position with BVM (disposable self-inflating resuscitator with a reservoir bag, O_2_ set at 15 L/min)	4-min preoxygenation in a head-up position with HFNC (60 L/min flow of headed and humidified oxygen FiO2 1.0, large or medium nasal cannulae chosen according to patients’ nostril size)	PaO_2_/ FiO_2_: BVM, 375 [276, 446] HFNC, 318 [242, 396]	Lowest SpO2 during intubation, SpO2 < 80%, aspiration, cardiac arrest, severe hypotension (SBP < 80 mmHg or vasopressor initiation or dose increment), 28-day mortality
Frat et al. [8] 2019	Twenty-eight ICUs in France	*N* = 313 Inclusion criteria: Patients (age > 18) admitted to the ICU requiring intubation, had acute hypoxemic respiratory failure (RR > 25 bpm or signs of respiratory distress, PaO_2_/FiO_2_ < 300 mmHg regardless of oxygenation strategy) Exclusion criteria: Cardiac arrest, altered consciousness (GCS < 8)	3–5-min preoxygenation at 30° with HFNC with oxygen flow 60 L/min through a heated humidifier, FiO_2_ 1.0. Clinicians performed a jaw thrust to maintain a patent upper airway, and continued high-flow oxygen therapy during laryngoscopy until endotracheal tube was placed into the trachea	3–5-min preoxygenation at 30° with NIV—pressure support ventilation delivered via a face mask connected to an ICU ventilator, adjusted to obtain an expired tidal volume 6–8 ml/kg of predicted body weight with PEEP 5 cmH_2_O and FiO_2_ 1.0	PaO_2_/FiO_2_: HFNC, 148 ± 70 NIV, 142 ± 65	Occurrence of an episode of severe hypoxemia (SpO_2_ < 80% for at least 5 s), lowest SpO_2_ during intubation, arterial hypotension, sustained arrhythmia, cardiac arrest, regurgitation, new infiltrate on chest radiography, 28-day mortality

*RR* respiratory rate, *bpm* breath per minute, *GCS* Glasgow coma scale, *RSI* rapid sequence induction, *NIV* noninvasive ventilation, *HFNC* high-flow nasal cannula, *PEEP* positive end-expiratory pressure, *BVM* bag-valve mask, *SBP* systolic blood pressure, *SOFA* Sequential Organ Failure Assessment, *SD* standard deviation, *IQR* interquartile range

### Risk of bias

**Fig. 2:**
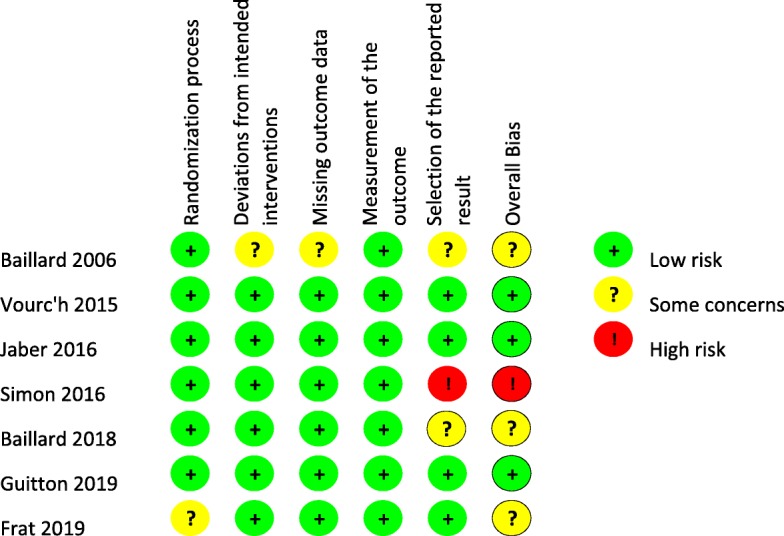
Risk of bias of included studies

The risk of bias was high in 1 trial, low in 3 trials, and with some concerns in 3 trials (Fig. 2).

### Quality of evidence

Direct comparisons often suffered from imprecision and limitations of risk of bias. There were no significant concerns regarding intransitivity. There was no significant incoherence detected by statistical testing nor visual inspection of direct and indirect estimates (Table 2).

**Table 2:** Direct, indirect, and network meta-analysis estimates of the effects of different preoxygenation methods

Comparison	No. of trials	Direct estimate (95% CI)	Quality	Indirect estimate (95% CI)	Quality^e^	NMA estimate (95% CI)	Quality
Lowest SpO_2_ during intubation (MD)							
HFNC vs. COT	3	− 1.64 (− 4.53, 1.25)	High	− 2.95 (− 8.23, 2.32)	Low^f,g^	− 1.94 (− 4.48, 0.59)	High
NIV vs. COT ^a^	2	− 5.95 (− 9.38, − 2.53)	Moderate^b^	− 4.64 (− 9.58, 0.31)	Moderate^f^	− 5.53 (− 8.34, − 2.71)	Moderate
HFNC vs. NIV	1	3.00 (− 1.01, 7.01)	Low^b,c^	4.31 (− 0.17, 8.80)	Moderate^f^	3.58 (0.59, 6.57)	Moderate
HFNC and NIV vs. NIV	1	− 3.10 (− 11.18, 4.98)	Moderate^b^	Not estimable^k^	–	− 3.10 (− 11.18, 4.98)	Moderate
SpO_2_ < 80% during intubation (OR)							
HFNC vs. COT	3	0.79 (0.32, 1.94)	Moderate^c^	0.44 (0.10, 1.95)	Very low^f,h^	0.67 (0.31, 1.46)	Moderate
NIV vs. COT	2	0.35 (0.13, 0.96)	Moderate^b^	0.63 (0.15, 2.60)	Moderate^f^	0.43 (0.19, 0.97)	Moderate
HFNC vs. NIV	1	1.25 (0.42, 3.75)	Moderate^c^	2.23 (0.58, 8.60)	Low^f,g^	1.58 (0.67, 3.69)	Moderate
HFNC and NIV vs. NIV	1	0.16 (0.01, 1.80)	Moderate^c^	Not estimable^k^		0.16 (0.01, 1.80)	Moderate
Intubation-related complications^i^ (OR)							
HFNC vs. COT	3	0.50 (0.27, 0.92)	High	0.44 (0.08, 2.53)	Low^j^	0.49 (0.28, 0.88)	High
NIV vs. COT	2	0.38 (0.07, 2.06)	Very low^b,d^	0.44 (0.20, 0.96)	Moderate^g^	0.43 (0.21, 0.87)	Moderate
HFNC vs. NIV	1	1.15 (0.70, 1.87)	Moderate^c^	1.30 (0.22, 7.77)	Very low^j,g^	1.16 (0.72, 1.86)	Moderate
HFNC and NIV vs. NIV	1	1.20 (0.31, 4.61)	Low^d^	Not estimable^k^		1.20 (0.31, 4.61)	Low
Mortality (OR)							
HFNC vs. COT	2	0.90 (0.55, 1.46)	High	0.58 (0.29, 1.17)	Low^f,g^	0.78 (0.52, 1.16)	High
NIV vs. COT	2	0.68 (0.40, 1.14)	Moderate^b^	1.04 (0.53, 2.04)	Low^f,g^	0.79 (0.53, 1.20)	Moderate
HFNC vs. NIV	1	0.86 (0.54, 1.37)	Moderate^b^	1.32 (0.65, 2.70)	Low^f,g^	0.98 (0.66, 1.45)	Moderate
HFNC and NIV vs. NIV	1	0.78 (0.24, 2.55)	Low^d^	Not estimable^k^	–	0.78 (0.24, 2.55)	Low

*COT* conventional oxygen therapy (bag-valve mask or facial mask), *HFNC* high-flow nasal cannula, *NIV* noninvasive ventilation, *MD* mean difference, *OR* odds ratio, *NMA* network meta-analysis

^a^The median and interquartile range of lowest SpO2 extracted from Baillard et al. [5] were converted to mean and standard deviation using a published equation [8]

^b^Quality of evidence for direct estimate rated down by one level for risk of bias

^c^Quality of evidence for direct estimate rated down by one level for serious imprecision

^d^Quality of evidence for direct estimate rated down by two levels for very serious imprecision

^e^We did not downgrade for intransitivity in indirect comparisons

^f^Quality of evidence for indirect estimate rated down by one level for serious imprecision

^g^Quality of evidence for indirect estimate rated down by one level for risk of bias

^h^Quality of evidence for indirect estimate rated down by two levels for serious risk of bias

^i^Intubation-related complications were defined as aspiration or new infiltrate on post-intubation chest radiograph, hemodynamic instability, and cardiac arrest

^j^Quality of evidence for indirect estimate rated down by two levels for very serious imprecision

^k^Cannot be estimated because it was not connected in a loop in the evidence network

### Clinical outcomes

#### Lowest SpO_2_ during intubation

**Fig. 3:**
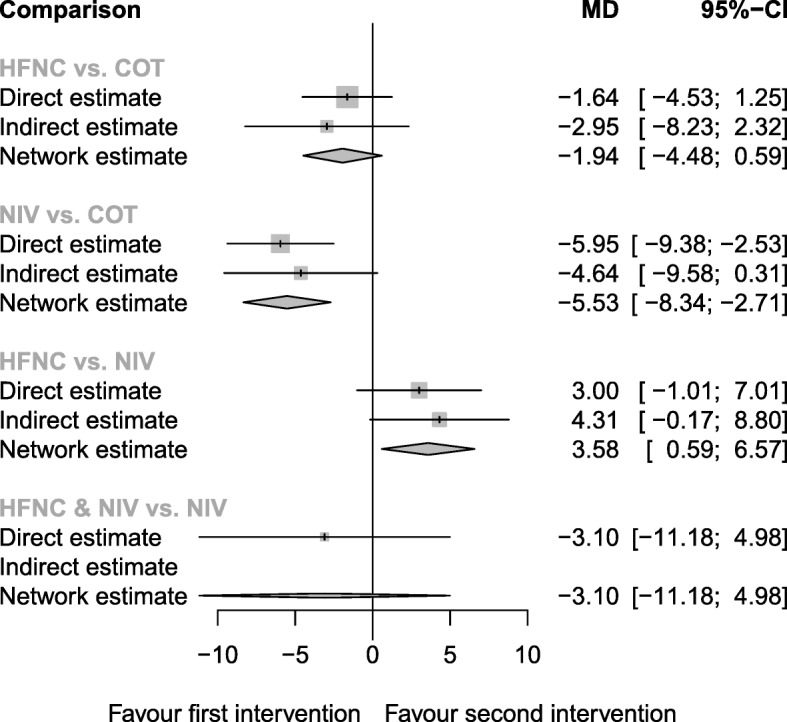
Forest plot of lowest SpO_2_ during intubation. *I*^2^ = 23.6%. *Q-*statistics for heterogeneity (within designs) and inconsistency (between designs). Total: *p* = 0.264, within designs: *p* = 0.162, between designs: *p* = 0.750. COT, conventional oxygen therapy (bag-valve mask or facial mask); HFNC, high-flow nasal cannula; NIV, noninvasive ventilation; MD, mean difference; NMA, network meta-analysis

Seven trials (959 patients) reported the lowest SpO_2_ during intubation (Additional file 2: Table S2). The network geometry was shown in Additional file 2: Figure S2. The network estimate provided high-to-moderate quality evidence (Table 2). Patients preoxygenated with NIV had significantly less desaturation than patients treated with COT (network estimate, MD 5.53, 95% CI 2.71, 8.34) and HFNC (network estimate, MD 3.58, 95% CI 0.59, 6.57) (Fig. 3). Although HFNC and NIV was ranked to be the best treatment (Table 3), there was no evidence that HFNC and NIV was superior than NIV (direct estimate, MD − 3.10, 95% CI − 11.18, 4.98), and thus, the result should be interpreted with caution. NIV ranked the 2nd among the four methods. The heterogeneity and consistency were low (*I*^2^ = 23.6%; *Q* statistics total: *p* = 0.264, within design: *p* = 0.162, between designs: *p* = 0.750).

**Table 3:** The *P*-score statistics

	HFNC and NIV	NIV	HFNC	COT
Lowest SpO_2_ during intubation	0.895	0.739	0.336	0.030
SpO_2_ < 80% during intubation	0.957	0.634	0.344	0.066
Intubation-related complications	0.560	0.774	0.595	0.071
Mortality	0.689	0.556	0.598	0.157

*P*-scores represent the extent of certainty that a treatment is better than the other treatments—the *P*-score would be close to 1 when a treatment is certain to be the best and close to 0 when a treatment is certain to be the worst

*COT* conventional oxygen therapy (bag-valve mask or facial mask), *HFNC* high-flow nasal cannula, *NIV* noninvasive ventilation, *MD* mean difference, *OR* odds ratio, *NMA* network meta-analysis

#### SpO_2_ < 80% during intubation

**Fig. 4:**
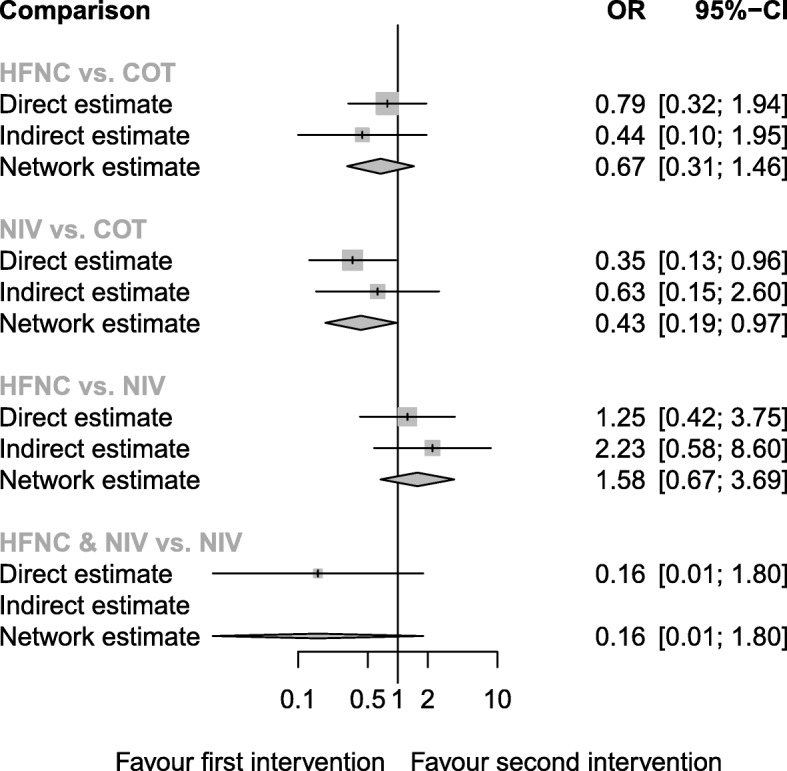
Forest plot of SpO_2_ < 80% during intubation. *I*^2^ = 48%. *Q*-statistics for heterogeneity (within designs) and inconsistency (between designs). Total: *p* = 0.104, within designs: *p* = 0.072, between designs: *p* = 0.409. COT, conventional oxygen therapy (bag-valve mask or facial mask); HFNC, high-flow nasal cannula; NIV, noninvasive ventilation; OR, odds ratio; NMA, network meta-analysis

Seven trials (959 patients) reported the incidence of SpO_2_ < 80% during intubation (Additional file 2: Table S2). The network estimate provided moderate-quality evidence (Table 2). Significant desaturation with SpO_2_ < 80% was less common in patients preoxygenated with NIV than that with COT (network estimate OR 0.43, 95%CI 0.19, 0.97) (Fig. 4). The combined use of HFNC and NIV was ranked to be the best preoxygenation method. However, the confidence interval of its treatment effect estimates was very wide because of the small sample size (direct estimate OR 0.16, 95% CI 0.01, 1.80). NIV ranked the 2nd among the four preoxygenation methods (Table 3).

#### Intubation-related complications

**Fig. 5:**
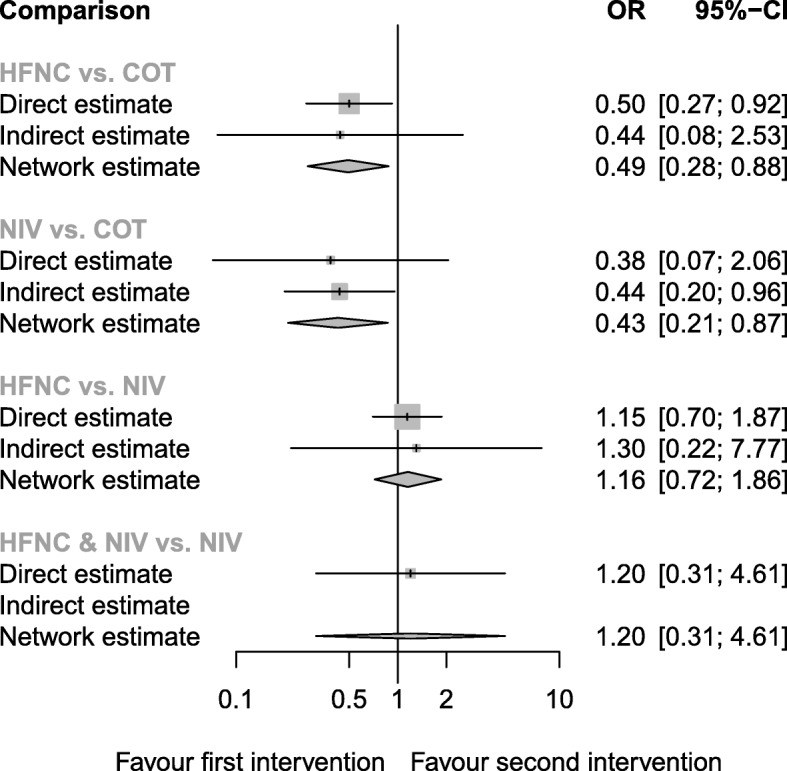
Forest plot of intubation-related complications. *I*^2^ = 0%. *Q*-statistics for heterogeneity (within designs) and inconsistency (between designs). Total: *p* = 0.978, within designs: *p* = 0.914, between designs: *p* = 0.892. Intubation-related complications were defined as aspiration or new infiltrate on post-intubation chest radiograph, hemodynamic instability, and cardiac arrest. COT, conventional oxygen therapy (bag-valve mask or facial mask); HFNC, high-flow nasal cannula; NIV, noninvasive ventilation; OR, odds ratio; NMA, network meta-analysis

Seven trials (959 patients) reported the intubation-related complications) (Additional file 2: Table S3). The network estimate ranged from high- to low-quality evidence (Table 2). Both NIV (network estimate OR 0.43, 95% CI 0.21, 0.87) and HFNC (network estimate OR 0.49, 95% CI 0.28, 0.88) resulted in a lower risk of intubation-related complications than COT (Fig. 5). NIV ranked the 1st and HFNC 2nd among the four preoxygenation methods (Table 3).

#### Mortality

**Fig. 6:**
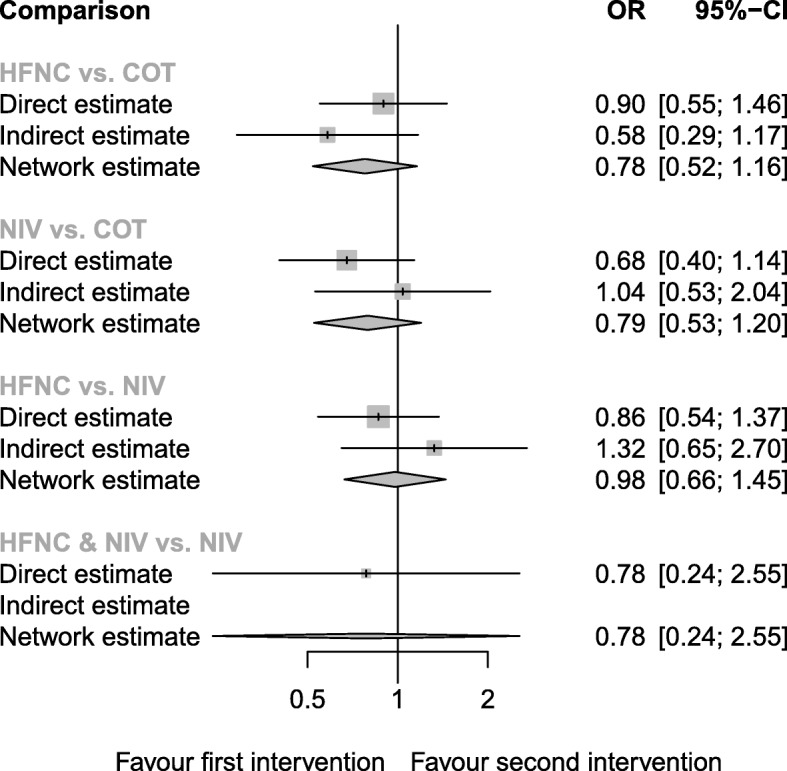
Forest plot of mortality. *I*^2^ = 0%. *Q*-statistics for heterogeneity (within designs) and inconsistency (between designs). Total: *p* = 0.533, within designs: *p* = 0.545, between designs: *p* = 0.322. COT, conventional oxygen therapy (bag-valve mask or facial mask); HFNC, high-flow nasal cannula; NIV, noninvasive ventilation; OR, odds ratio; NMA, network meta-analysis

Six trials (919 patients) reported the mortality (Additional file 2: Table S2). The network estimate ranged from high- to low-quality evidence (Table 2): There was no evidence showing the superiority of one particular preoxygenation method as determined by the confidence interval (Fig. 6). Thus, the ranking by *P*-scores should be interpreted with caution (Table 3).

### Sensitivity analysis

We performed sensitivity analysis by excluding the study by Guitton et al. [5] as subjects with mild hypoxemia were included, compared with the other included studies which recruited subjects with moderate to severe respiratory failure. Results of direct, indirect, and network estimates were similar (Additional file 2: Figures S3–S6). There was no change in ranking based on *P*-scores (Additional file 2: Table S5).

## Discussion

In this network meta-analysis, we included 7 RCTs enrolling 959 patients comparing three preoxygenation methods—COT, HFNC, and NIV. Hypoxemic patients treated with NIV during intubation desaturated less (as reflected by absolute difference of lowest SpO_2_ and incidence of SpO_2_ < 80%) than those patients treated with HFNC or COT (moderate quality of evidence). The risk of intubation-related complications (aspiration or new infiltrate on postintubation chest radiograph, hemodynamic instability, and cardiac arrest) was lower with NIV than with any other preoxygenation methods (moderate quality of evidence). Among the methods of preoxygenation examined, it seems that combined use of HFNC and NIV is the most effective in minimizing the drop in SpO_2_ and the incidence of SpO_2_ < 80% during intubation. However, this determination is limited to data from only one single-center study in which no head-to-head comparisons were performed against all other methods.

HFNC has several physiological advantages, including its ability to deliver high-flow oxygen, generation of low level of PEEP, and allowing apneic oxygenation [21]. Despite the clear benefit of HFNC in patients with acute hypoxemic respiratory failure and after planned extubation [22, 23], the evidence for HFNC in preoxygenation remains conflicting. The single-center before-after study by Miguel-Montanes et al. [24] has excluded patients with severe hypoxemia. Its positive results have not been reproducible in the subsequent trials by Vourc’h et al. [3] and Simon et al. [4] which recruited patients with severe hypoxemia and patients with mild-moderate hypoxemia respectively. While Guitton et al. [5] have shown a reduction in intubation-related adverse events with the use of HFNC in non-severely hypoxemic patient, it was not accompanied by an improvement in the lowest SpO_2_. The effectiveness of HFNC is undermined by the loss of PEEP effect due to mouth opening in patients in respiratory distress [25]. These patients can have a dramatic increase in inspiratory nasal and oral flow rate of up to 110 L/min and 280 L/min respectively, and that could not be matched by the HFNC [26]. Another possible explanation is that apneic oxygenation requires a continuous oxygen extraction from the functional residual capacity (FRC) during the apnea period, thus generating a pressure gradient to allow oxygen flow from the nose into the alveoli. These mechanisms are hindered by the reduction of FRC and shunt physiology in diseased lungs [27]. Airflow may also be limited by the use of cricoid pressure possibly obstructing the space between the oropharynx and trachea [28].

NIV allows the delivery of high level of FiO_2_ and positive intrathoracic pressure, encouraging alveolar recruitment which could possibly improve the efficiency of gaseous exchange. It has also been shown to counteract inward air leaks and improve face-mask seal [29]. The theoretical risk of gastric distention and aspiration remains unproven based on our analysis. Although the mask must be removed during laryngoscopy, patients preoxygenated with NIV still desaturated less during intubation than patients preoxygenated with other modalities. The authors thus recommend the use of NIV for preoxygenation in patients with acute hypoxemic respiratory failure.

The next question would be whether the addition of HFNC to NIV could produce extra benefit during preoxygenation. The pilot study by Jaber et al. [20] published in 2016 showed promising results. However, one must be cautious in the interpretation of the findings in the use of HFNC and NIV, in view of the small sample size that tends to overestimate the treatment effect and the possibility of publication bias. Additionally, the optimal way to minimize air leak with concomitant use of HFNC and NIV has not been well-delineated. It would be interesting to consider whether nasal continuous positive airway pressure mask could play a special role in preoxygenation. Visualization of glottic view by laryngoscopy may be feasible with the nasal mask in situ, maintaining oxygenation during intubation.

Another practical but unaddressed consideration would be the oxygen device used prior to the decision of intubation. Based on the mortality difference shown in FLORALI-1 trial, it would not be surprising to see a surge in the use of HFNC in patients with acute hypoxemic respiratory failure. A more recent systematic review also confirmed a reduced risk of requiring intubation with the use of HFNC compared with COT [22]. All existing studies placed no restriction in the patient enrollment based on the oxygen devices they used prior to inclusion and the number of patients on advanced oxygen device varied across studies (Additional file 2: Table S4). As shown in the study by Baillard et al. [7], there was an increased risk of adverse events including severe desaturation with SpO_2_ < 80% in patients initially put on NIV, who were then randomized to receive COT during preoxygenation. It would be uncertain whether the lowered complication risks during preoxygenation with NIV was in fact a reflection of a higher risk of complication in patients “de-escalated” from a more advanced oxygen support (NIV) to a simpler device (COT). Additionally, whether the advantage of oxygenation in NIV would be less evident in patients who were already put on NIV or HFNC remained unclear.

The study is the first systematic review using network meta-analysis to evaluate preoxygenation methods in patients with acute hypoxemic respiratory failure. NMA allows the comparison of multiple preoxygenation methods and increases the precision by combining direct and indirect estimates. Other strengths of this study included the comprehensive search, duplicate and independent citation screening and data abstraction, use of the latest modified Cochrane risk of bias assessment tool, and the adherence to the PRISMA-NMA guideline.

This study had some limitations. Despite an extensive literature search, the number of trials for each comparison of preoxygenation methods remained small. Funnel plot was not constructed because of the limited number of studies, so it is not possible to estimate possible publication bias. While pulse oximetry was frequently used as the outcome measure in clinical trials studying preoxygenation, it is, however, not the best surrogate to reflect systematic oxygenation [30]. Arterial oxygen saturation or end-tidal oxygen is more relevant to assess the adequacy of preoxygenation, but they may not be readily available in case of clinical emergency settings.

## Conclusions

In adult patients with acute hypoxemic respiratory failure, NIV is a safe and probably the most effective preoxygenation method. Further research should be performed to evaluate the benefits of the combination strategy of NIV plus HFNC.

## Supplementary information

**Additional file 1:** PRISMA checklist. (DOCX 23.5 kb)

**Additional file 2:** **Figure S1.** Timing of preoxygenation methods. **Figure S2.** Network of preoxygenation methods for evaluating lowest SpO_2._ The size of the nodes was proportional to the number of patients randomized to each preoxygenation methods and thickness of the lines to the number of direct comparisons. **Figure S3.**Sensitivity analysis of forest plot of lowest SpO_2_ during intubation. I^2^ = 42.6%. Q-statistics for heterogeneity (within designs) and inconsistency (between designs). Total: *p* = 0.156, within designs: *p* = 0.083, between designs: *p* = 0.616. **Figure S4.** Sensitivity analysis of forest plot of SpO_2_ < 80% during intubation. I^2^ = 0%. Q-statistics for heterogeneity (within designs) and inconsistency (between designs). Total: *p* = 0.574, within designs: *p* = 0.354, between designs: *p* = 0.615. **Figure S5.** Sensitivity analysis of forest plot of intubation-related complications. I^2^ = 0%. Q-statistics for heterogeneity (within designs) and inconsistency (between designs). Total: *p* = 0.933, within designs: *p* = 0.749, between designs: *p* = 0.848. **Figure S6.** Sensitivity analysis of forest plot of mortality. I^2^ = 0%. Q-statistics for heterogeneity (within designs) and inconsistency (between designs). Total: *p* = 0.574, within designs: *p* = 0.354, between designs: *p* = 0.615. **Table S1.** PubMED search strategy. **Table S2.** Lowest SpO_2_, incidence of SpO_2_ < 80% during intubation, and mortality. NR, not reported; SD, standard deviation. ^a^ICU mortality. ^b^28-day mortality. ^c^Only median and interquartile range were provided in the study: NIV 92 (84–98), COT 88 (79–95). Data were transformed into mean and standard deviation using a published equation [12]. **Table S3.** Intubation-related complications. NR, not reported; COT, conventional oxygen therapy (bag-valve mask or facial mask); HFNC, high-flow nasal cannula; NIV, noninvasive ventilation; ^a^Adverse events including cardiac arrest, hemodynamic instability and aspiration of gastric contents were included as outcome measure and it was reported that there were no adverse events related to intubation in the study. **Table S4.** Advanced oxygen devices used before study inclusion. NR, not reported; COT, conventional oxygen therapy (bag-valve mask or facial mask); HFNC, high-flow nasal cannula; NIV, noninvasive ventilation. **Table S5.** P-scores statistics of sensitivity analysis. (DOCX 840 kb)

## Data Availability

All data generated or analyzed during the present study are included in this published article and its supplementary information files.

## Abbreviations

COT: Conventional oxygen therapy
NIV: Noninvasive ventilation
HFNC: High-flow nasal cannula
ICU: Intensive care unit
PEEP: Positive end-expiratory pressure
NMA: Network meta-analysis
RCT: Randomized controlled trials
MD: Mean difference
OR: Odds ratio
CI: Confidence interval
NR: Not reported
RR: Respiratory rate
bpm: Breath per minute
GCS: Glasgow coma scale
RSI: Rapid sequence induction
BVM: Bag-valve mask
SBP: Systolic blood pressure
SOFA: Sequential Organ Failure Assessment
SD: Standard deviation
IQR: Interquartile range
