# Supplementary material for: Preoxygenation before intubation in adult patients with acute hypoxemic respiratory failure: a network meta-analysis of randomized trials
Source: Crit Care. 2019 Sep 18;23:319. doi: 10.1186/s13054-019-2596-1 (PMC6751657; doi:10.1186/s13054-019-2596-1)
Supplement: Supplementary file 2 — Additional file 2: Figure S1. Timing of preoxygenation methods. Figure S2. Network of preoxygenation methods for evaluating lowest SpO2. The size of the nodes was proportional to the number of patients randomized to each preoxygenation methods and thickness of the lines to the number of direct comparisons. Figure S3.Sensitivity analysis of forest plot of lowest SpO2 during intubation. I2 = 42.6%. Q-statistics for heterogeneity (within designs) and inconsistency (between designs). Total: p = 0.156, within designs: p = 0.083, between designs: p = 0.616. Figure S4. Sensitivity analysis of forest plot of SpO2 < 80% during intubation. I2 = 0%. Q-statistics for heterogeneity (within designs) and inconsistency (between designs). Total: p = 0.574, within designs: p = 0.354, between designs: p = 0.615. Figure S5. Sensitivity analysis of forest plot of intubation-related complications. I2 = 0%. Q-statistics for heterogeneity (within designs) and inconsistency (between designs). Total: p = 0.933, within designs: p = 0.749, between designs: p = 0.848. Figure S6. Sensitivity analysis of forest plot of mortality. I2 = 0%. Q-statistics for heterogeneity (within designs) and inconsistency (between designs). Total: p = 0.574, within designs: p = 0.354, between designs: p = 0.615. Table S1. PubMED search strategy. Table S2. Lowest SpO2, incidence of SpO2 < 80% during intubation, and mortality. NR, not reported; SD, standard deviation. aICU mortality. b28-day mortality. cOnly median and interquartile range were provided in the study: NIV 92 (84–98), COT 88 (79–95). Data were transformed into mean and standard deviation using a published equation [12]. Table S3. Intubation-related complications. NR, not reported; COT, conventional oxygen therapy (bag-valve mask or facial mask); HFNC, high-flow nasal cannula; NIV, noninvasive ventilation; aAdverse events including cardiac arrest, hemodynamic instability and aspiration of gastric contents were included as outcome measure and it was repor [file 13054_2019_2596_MOESM2_ESM.docx]

**Figure S1. Timing of preoxygenation methods**

**
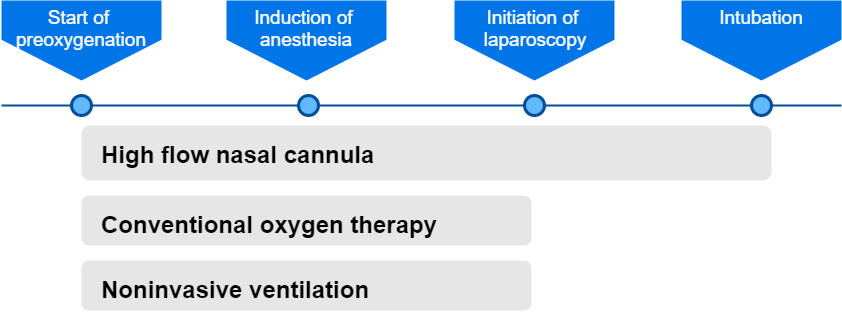
**

**Figure S2. Network of preoxygenation methods for evaluating lowest SpO_2_**


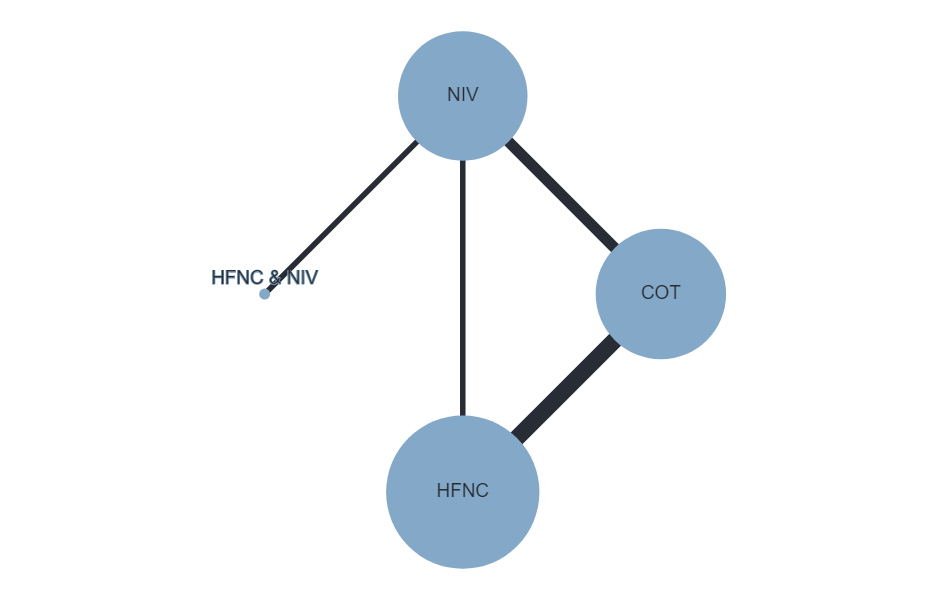


The size of the nodes was proportional to the number of patients randomized to each preoxygenation methods and thickness of the lines to the number of direct comparisons.

**Figure S3. Sensitivity analysis of forest plot of lowest SpO_2_ during intubation**


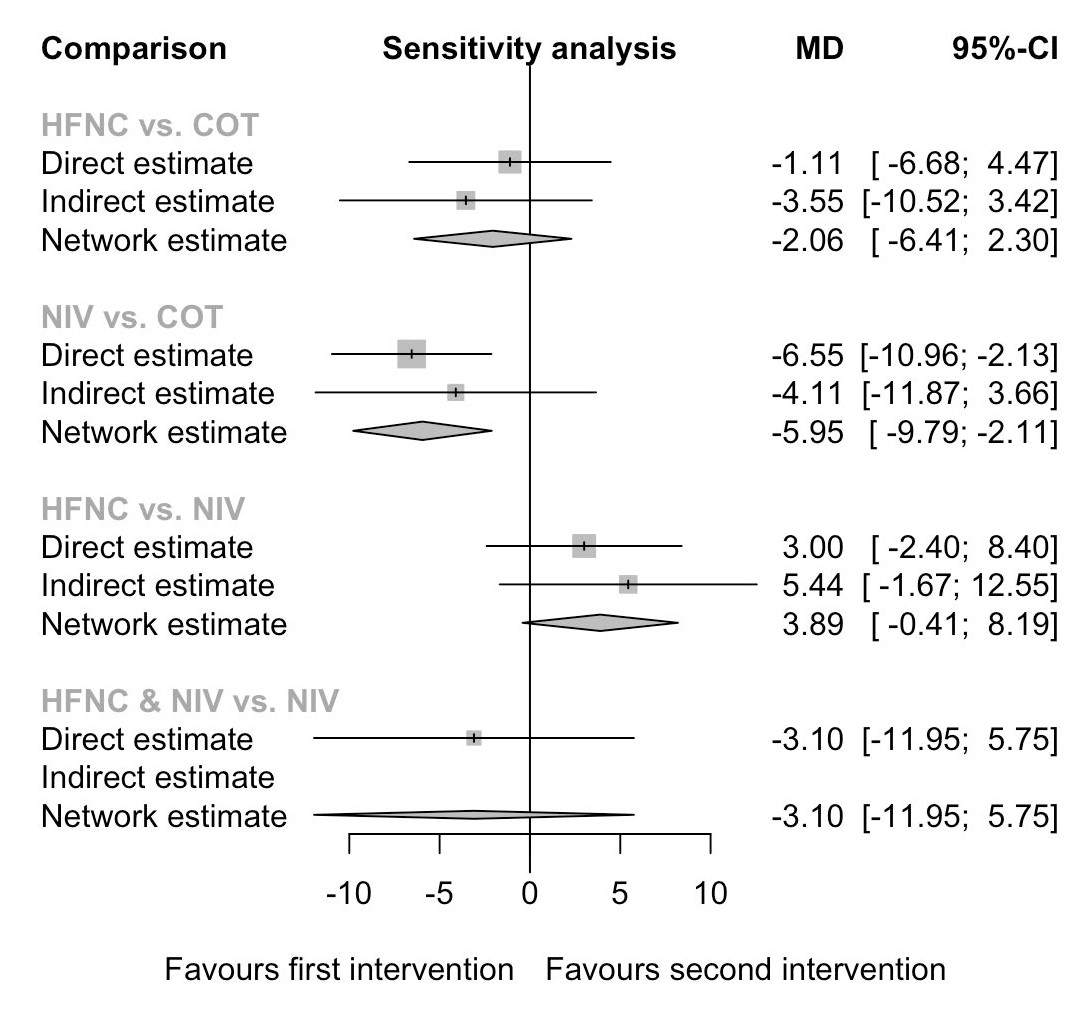


I^2^ = 42.6%

Q-statistics for heterogeneity (within designs) and inconsistency (between designs)

Total: *p*=0.156, within designs: *p*=0.083, between designs: *p*=0.616

**Figure S4. Sensitivity analysis of forest plot of SpO_2_<80% during intubation**


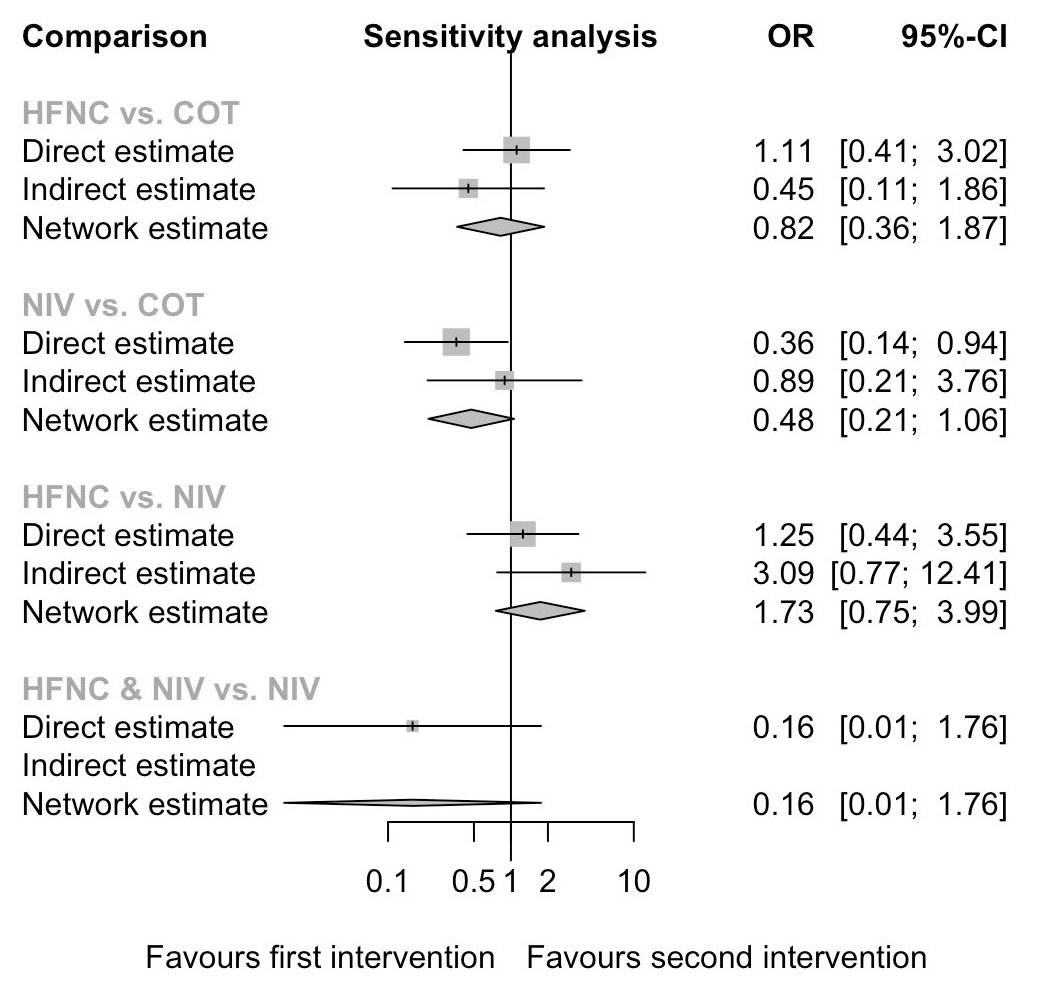


I^2^ = 0%

Q-statistics for heterogeneity (within designs) and inconsistency (between designs)

Total: *p*=0.574, within designs: *p*=0.354, between designs: *p*=0.615

**Figure S5. Sensitivity analysis of forest plot of intubation-related complications**


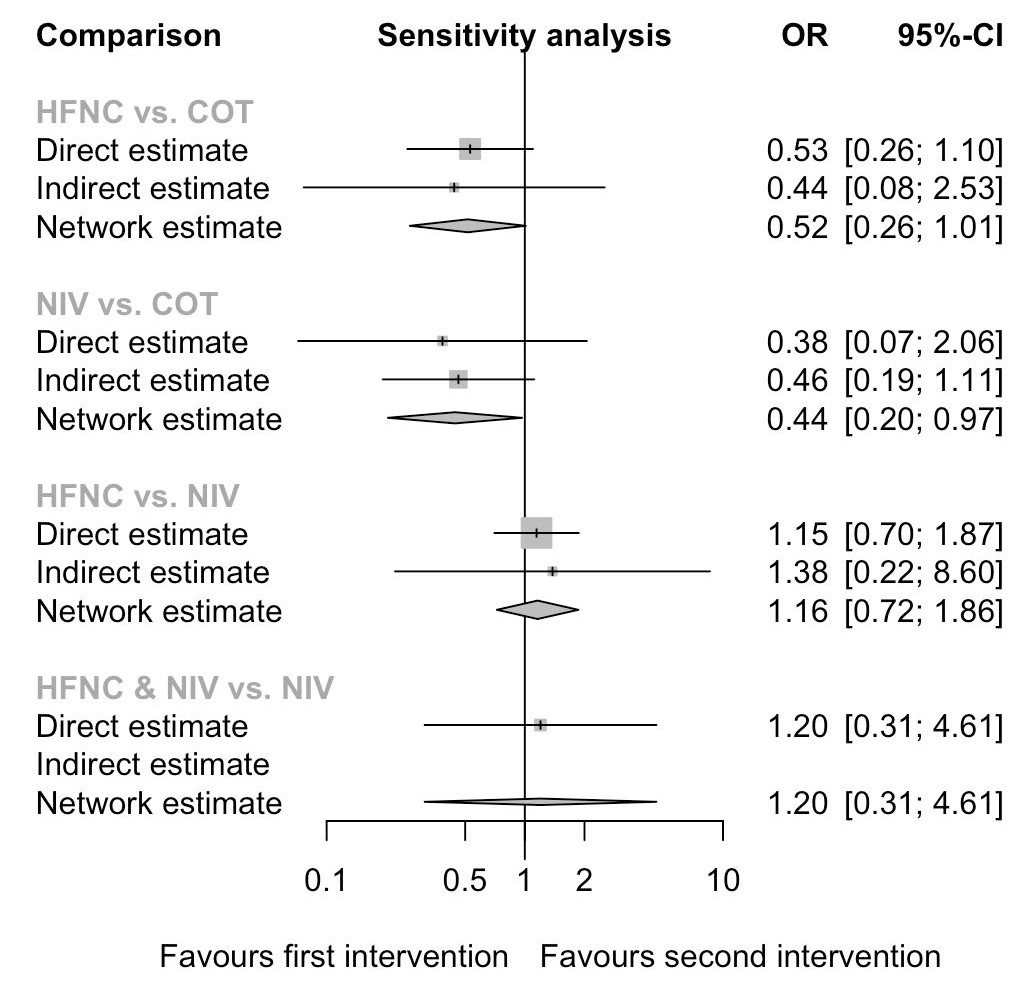


I^2^ = 0%

Q-statistics for heterogeneity (within designs) and inconsistency (between designs)

Total: *p*=0.933, within designs: *p*=0.749, between designs: *p*=0.848

**Figure S6. Sensitivity analysis of forest plot of mortality**


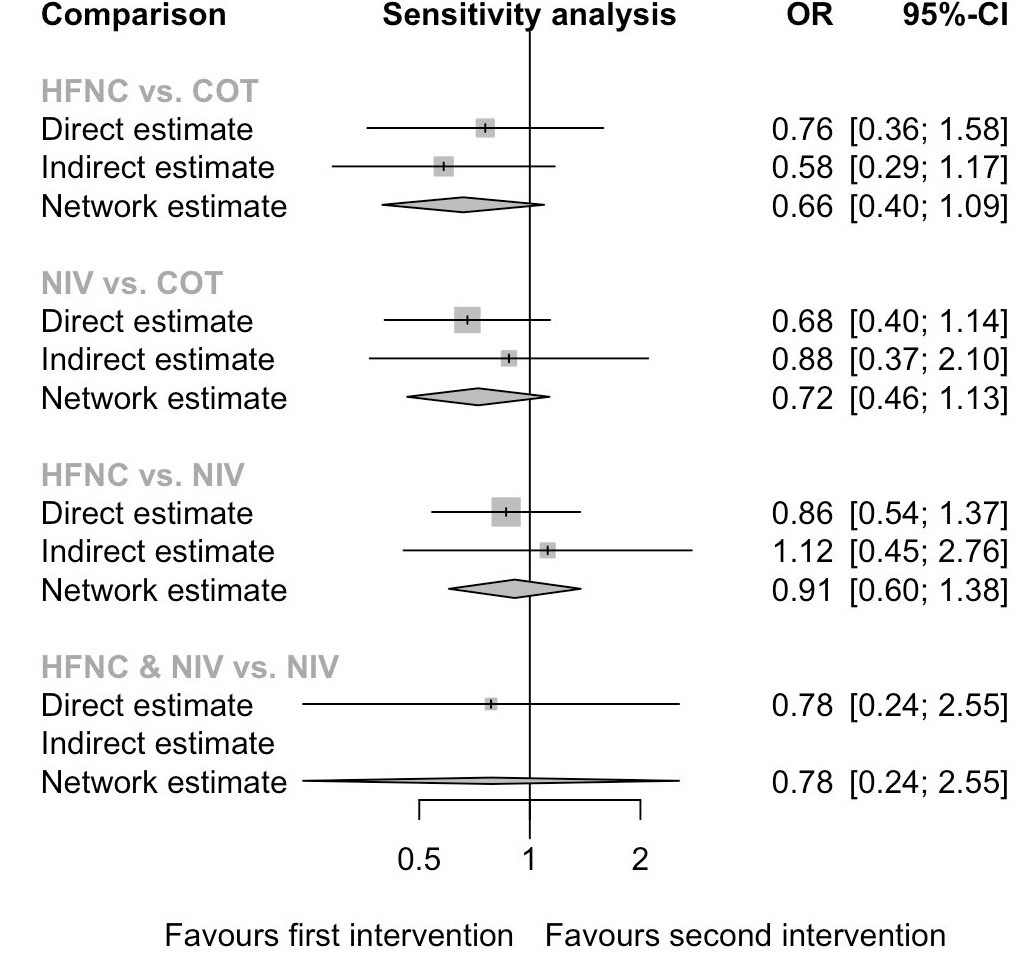


I^2^ = 0%

Q-statistics for heterogeneity (within designs) and inconsistency (between designs)

Total: *p*=0.574, within designs: *p*=0.354, between designs: *p*=0.615

**Table S1. PubMED search strategy**

| # | Searches |
| --- | --- |
| 1 | (hypox*) OR respiratory failure) OR ((intensive care) OR ICU OR critical OR emergency) |
| 2 | ((((high flow) or (high-flow)) AND (nasal OR oxygen))) OR apn*ic oxygenation) OR ((bag valve mask) OR (non rebreath*))) OR ((noninvasive OR (non invasive) OR (non-invasive)) AND ventilation |
| 3 | intubation OR preoxygenation OR (pre oxygenation) |
| 4 | (((clinical trials as topic[MeSH Terms]) OR randomized controlled trial[Publication Type]) OR controlled clinical trial[Publication Type]) OR trial [Title/Abstract]) OR random [Title/Abstract] |
| 5 | #1 AND #2 AND #3 AND #4 |

**Table S2. Lowest SpO_2_, incidence of SpO_2_ <80% during intubation, and mortality**

|  |  |  | First intervention | | |  |  | Second intervention | | |
| --- | --- | --- | --- | --- | --- | --- | --- | --- | --- | --- |
|  | First intervention | n | Lowest SpO_2_ during intubation,  mean±SD | Incidence of SpO_2_ < 80% during intubation,  n (%) | Mortality,  n (%) | Second intervention | n | Lowest SpO_2_ during intubation,  mean±SD | Incidence of SpO_2_ < 80% during intubation,  n (%) | Mortality,  n (%) |
| Baillard et al. [6] | COT | 26 | 81 ± 5% | 12 (46.2) | 13 (50)^a^ | NIV | 27 | 93 ± 8% | 2 (7.4) | 8 (29.6)^a^ |
| Vourc’h et al. [3] | COT | 57 | 85.9 ± 12.9% | 13 (22.8) | 24 (42.1)^b^ | HFNC | 62 | 86.2 ± 15.2% | 16 (25.8) | 22 (35.5)^b^ |
| Jaber et al.  [20] | HFNC and NIV | 25 | 94.6 ± 15.0% | 1 (4.0) | 8 (32.0)^b^ | NIV | 24 | 91.5 ± 12.5% | 5 (20.8) | 9 (37.5)^b^ |
| Simon et al.^a^ [4] | COT | 20 | 86 ± 11% | 5 (25.0) | NR | HFNC | 20 | 89 ± 18% | 5 (25.0) | NR |
| Baillard et al. [7] | COT | 102 | 87.33 ± 12.03%^c^ | 28 (27.5) | 38 (37.3)^b^ | NIV | 99 | 91.33±10.53%^c^ | 18 (18.2) | 31 (31.3)^b^ |
| Guitton et al. [5] | COT | 89 | 95 ± 12% | 7 (7.9) | 24 (27.0)^b^ | HFNC | 95 | 97 ± 5% | 2 (2.1) | 26 (27.4)^b^ |
| Frat et al. [8] | HFNC | 171 | 84 ± 16% | 47 (27.5) | 58 (33.9)^b^ | NIV | 142 | 87 ± 13% | 33 (23.2) | 53 (37.3)^b^ |

NR, not reported; SD, standard deviation

^a^ICU mortality

^b^28-day mortality

^c^Only median and interquarile range were provided in the study: NIV 92 (84-98), COT 88 (79-95). Data were transformed into mean and standard deviation using a published equation [12].

**Table S3. Intubation-related complications**

|  |  |  | First intervention | | |  |  | Second intervention | | |
| --- | --- | --- | --- | --- | --- | --- | --- | --- | --- | --- |
|  | First intervention | n | Aspiration/ new infiltrate on post-intubation chest radiograph, n(%) | Haemodynamic instability, n(%) | Cardiac arrest, n(%) | Second intervention | n | Aspiration/ new infiltrate on post-intubation chest radiograph, n(%) | Haemodynamic instability, n(%) | Cardiac arrest, n(%) |
| Baillard et al. [6] | COT | 26 | 3 (11.5) | NR | NR | NIV | 27 | 1 (3.7) | NR | NR |
| Vourc’h et al. [3] | COT | 57 | 0 | 30 (52.6) | 1 (1.6) | HFNC | 62 | 0 | 24 (38.7) | 0 |
| Jaber et al. [20] | HFNC and NIV | 25 | 0 | 6 (24.0) | 0 | NIV | 24 | 0 | 5 (20.8) | 0 |
| Simon et al.^a^ [4] | COT | 20 | 0 | 0 | 0 | HFNC | 20 | 0 | 0 | 0 |
| Baillard et al. [7] | COT | 102 | 0 | 2 (2.0) | NR | NIV | 99 | 0 | 1 (1.0) | NR |
| Guitton et al. [5] | COT | 89 | 2 (2.2) | 8 (9.0) | 0 | HFNC | 95 | 0 | 4 (4.2) | 1 (1.1) |
| Frat et al. [8] | HFNC | 171 | 33 (19.3) | 86 (50.3) | 5 (2.9) | NIV | 142 | 28 (19.7) | 70 (49.3) | 1 (0.7) |

NR, not reported; COT, conventional oxygen therapy (bag-valve mask or facial mask); HFNC, high-flow nasal cannula; NIV, noninvasive ventilation;

^a^Adverse events including cardiac arrest, haemodynamic instability and aspiration of gastric contents were included as outcome measure and it was reported that there were no adverse events related to intubation in the study.

**Table S4. Advanced oxygen devices used before study inclusion**

|  |  |  | Oxygen devices | |  |  | Oxygen devices | |
| --- | --- | --- | --- | --- | --- | --- | --- | --- |
|  | First intervention | n | NIV, n (%) | HFNC, n (%) | Second intervention | n | NIV, n (%) | HFNC, n (%) |
| Baillard et al. [6] | COT | 26 | NR | NR | NIV | 27 | NR | NR |
| Vourc’h et al. [3] | COT | 57 | 8 (14) | 3 (5.3) | HFNC | 62 | 11 (17.8) | 10 (16.1) |
| Jaber et al. [20] | HFNC and NIV | 25 | 7 (28) | 11 (33) | NIV | 24 | 8 (33) | 11 (46) |
| Simon et al.^a^ [4] | COT | 20 | 2 (10) | 1 (5) | HFNC | 20 | 3 (15) | 5 (25) |
| Baillard et al. [7] | COT | 102 | 46 (45.1) | NR | NIV | 99 | 45 (45.5) | NR |
| Guitton et al. [5] | COT | 89 | 8 (9.0) | 2 (2.2) | HFNC | 95 | 6 (6.3) | 3 (3.2) |
| Frat et al. [8] | HFNC | 171 | 41(24.0) | 57 (33.3) | NIV | 142 | 31(21.8) | 48 (3.8) |

NR, not reported; COT, conventional oxygen therapy (bag-valve mask or facial mask); HFNC, high-flow nasal cannula; NIV, noninvasive ventilation;

**Table S5 P-scores statistics of sensitivity analysis**

|  | HFNC & NIV | NIV | HFNC | COT |
| --- | --- | --- | --- | --- |
| Lowest SpO_2_ during intubation | 0.880 | 0.736 | 0.314 | 0.070 |
| SpO_2_ < 80% during intubation | 0.959 | 0.645 | 0.270 | 0.126 |
| Intubation-related complications | 0.554 | 0.772 | 0.587 | 0.087 |
| Mortality | 0.687 | 0.531 | 0.676 | 0.107 |
